# *ABCD2* Is a Direct Target of *β*-Catenin and TCF-4: Implications for X-Linked Adrenoleukodystrophy Therapy

**DOI:** 10.1371/journal.pone.0056242

**Published:** 2013-02-21

**Authors:** Chul-Yong Park, Han-Soo Kim, Jiho Jang, Hyunji Lee, Jae Souk Lee, Jeong-Eun Yoo, Dongjin R. Lee, Dong-Wook Kim

**Affiliations:** 1 Department of Physiology, Yonsei University College of Medicine, Seodaemun-gu, Seoul, Korea; 2 Severance Biomedical Research Institute, Yonsei University College of Medicine, Seoul, Korea; 3 Department of Laboratory Medicine and Cell Therapy Center, Yonsei University College of Medicine, Seoul, Korea; 4 Brain Korea 21 Project for Medical Science, Yonsei University College of Medicine, Seoul, Korea; Northwestern University Feinberg School of Medicine, United States of America

## Abstract

X-linked adrenoleukodystrophy (X-ALD) is a peroxisomal disorder caused by mutations in the *ABCD1* gene that encodes the peroxisomal ATP-binding cassette (ABC) transporter subfamily D member 1 protein (ABCD1), which is referred to as the adrenoleukodystrophy protein (ALDP). Induction of the *ABCD2* gene, the closest homolog of *ABCD1*, has been mentioned as a possible therapeutic option for the defective ABCD1 protein in X-ALD. However, little is known about the transcriptional regulation of *ABCD2* gene expression. Here, through in silico analysis, we found two putative TCF-4 binding elements between nucleotide positions −360 and −260 of the promoter region of the *ABCD2* gene. The transcriptional activity of the *ABCD2* promoter was strongly increased by ectopic expression of *β*-catenin and TCF-4. In addition, mutation of either or both TCF-4 binding elements by site-directed mutagenesis decreased promoter activity. This was further validated by the finding that *β*-catenin and the promoter of the *ABCD2* gene were pulled down with a *β*-catenin antibody in a chromatin immunoprecipitation assay. Moreover, real-time PCR analysis revealed that *β*-catenin and TCF-4 increased mRNA levels of *ABCD2* in both a hepatocellular carcinoma cell line and primary fibroblasts from an X-ALD patient. Interestingly, we found that the levels of very long chain fatty acids were decreased by ectopic expression of ABCD2-GFP as well as *β*-catenin and TCF-4. Taken together, our results demonstrate for the first time the direct regulation of *ABCD2* by *β*-catenin and TCF-4.

## Introduction

X-linked adrenoleukodystrophy (X-ALD) is the most common peroxisomal disorder and is caused by mutations or large deletions of one or more exons in the *ABCD1* gene located in Xq28, which encodes the peroxisomal member of the ATP-binding cassette (ABC) transporter subfamily D member 1 (ABCD1), also known as adrenoleukodystrophy protein (ALDP) [1], [2]. X-ALD has an incidence of 1 in 17,000 males, and has several clinical phenotypes, namely severe childhood cerebral form (CCALD, early-onset type), a slowly progressive form called adrenomyeloneuropathy (AMN, late-onset type), and adult cerebral form (ACALD) [3], [4]. Currently, no therapeutic drugs for X-ALD are available, although gene correction of autologous hematopoietic stem cell with a wild-type version of the *ABCD1* gene by a lentiviral vector has been shown to provide clinical benefit in X-ALD patients [5]. ABCD1 transports very long chain fatty acids (VLCFAs; those with more than 22 carbon atoms) or their CoA derivatives across the peroxisomal membrane for *β*-oxidation. Recently, it was demonstrated that human ABCD1 was able to transport VLCFA-CoA into the peroxisome in a yeast system [6]. Dysfunction of ABCD1 results in increased levels of saturated (C24∶0 and C26∶0) and monounsaturated (C26∶1) VLCFAs in the plasma and tissues of X-ALD patients, due to the reduced *β*-oxidation of VLCFAs in peroxisomes [7]. Recently, we first reported the generation of X-ALD patient-derived induced pluripotent stem (iPS) cell models [8]. Generated X-ALD iPS cells were successfully differentiated into oligodendrocytes, the main cell type affected by the disease, and notably revealed the underlying pathophysiology which had not been observed in patients’ fibroblasts or animal models.

*ABCD2* encodes the ALDP-related protein (ALDRP or ABCD2) that share high homology with ABCD1 implying their functional redundancy or functional overlap [9]. Indeed, overexpression of ABCD2 has been shown to normalize peroxisomal *β*-oxidation and prevent accumulation of VLCFAs in cultured human fibroblast cells obtained from an X-ALD patient [10]–[12] and in an *Abcd1* knockout mice model [13]. In addition, it has been reported that levels of VLCFAs are restored by pharmacological induction of the *ABCD2* gene [10], [14], [15]. Thus, this complementing gene can be an attractive target for X-ALD therapy [16].

*β*-catenin is not only a key protein involved in cell-cell adhesion complexes with E-cadherin, but also an important component of the Wnt signaling pathway, which is involved in diverse cellular processes including cell growth, migration, differentiation, gene transcription, development of the nervous system, and self-renewal of stem cells [17]–[20]. In the presence of Wnt signaling, cytoplasmic *β*-catenin translocates into the nucleus [21] where it forms a transcription complex with the lymphoid enhancer factor/T-cell factor (LEF/TCF) DNA binding protein, resulting in the transcription of target genes [22]. There are four TCF proteins (TCF-1, LEF-1, TCF-3, TCF-4) in mammalian cells [23]. In the absence of Wnt signaling, the TCFs interact with transcriptional repressors (such as Groucho) and gene expression of target genes is inhibited [24].

Weinhofer *et al*., via promoter studies, demonstrated that sterol regulatory element-binding proteins (SREBPs), a family of transcription factors that control the metabolism of cholesterol and fatty acids, are involved in transcription of the *ABCD2* gene [25], [26]. However, the identities of other transcription factors involved in transcription of the *ABCD2* gene are not known. In this study, we investigated putative transcription factors affecting the expression of *ABCD2* gene through in silico analysis, and found two putative LEF-1/TCF binding elements between nucleotide positions −360 and −260 of the promoter of the *ABCD2* gene. To our knowledge, this is the first report that *β*-catenin and TCF-4 directly regulate the expression of *ABCD2*; this direct regulation may provide a new drug discovery strategy for X-ALD.

## Materials and Methods

### Cell Cultures

HepG2 cells (human hepatocellular carcinoma cell line) were cultured in RPMI1640 medium (Sigma) supplemented with 10% fetal bovine serum (FBS) and 1% penicillin/streptomycin (Invitrogen). Huh7 cells (human hepatoma cell line) were cultured in Dulbecco’s modified Eagle’s medium (DMEM) supplemented with 10% FBS and 1% antibiotics. Human X-ALD fibroblasts from a single patient with X-ALD (CCALD type; GM04496, Coriell Institute) were cultured in Eagle’s minimumessential medium (MEM, Invitrogen) supplemented with 15% FBS and 1% antibiotics. Human dermal fibroblast (HDF) cells were purchased from Invitrogen (#C-004-5C) and were cultured as recommended by the supplier.

### Plasmids, Reporter Gene Constructs, and Site-directed Mutagenesis

Expression vectors for Flag-tagged constitutively active *β*-catenin (*β*-catenin 4A; S33A, S37A, T41A, S45A), Myc-tagged wild-type TCF-4, and dominant negative TCF-4 (lacking the *β*-catenin binding site; DN-TCF-4) were obtained from Addgene (plasmid #24204, #16514, #24310, respectively; Cambridge CA) [27], [28]. The human ABCD2-GFP expression vector was purchased from OriGene (#RG211199). Human *ABCD2* promoter fragments (−1300, −800, −500, −1300/−500, −360, −260, −160 bp) were amplified by PCR using *i*-MAX™ II DNA polymerase (iNtRON biotechnology, Korea) from HDF genomic DNA as a template, and subcloned into the pGL3-basic (Promega) vector. The primer sequences containing restriction sites are listed in Table 1. The *ABCD2* promoter sequence (starting from −1300 bp) was aligned with the sequence of human chromosome 12 (NCBI, Gene ID: 225). Mutations in the LEF-1/TCF binding sites were introduced using the 800-luc reporter plasmid by overlap extension PCR as described previously [29]. In brief, PCRs were performed to generate two DNA fragments (5′ fragment and 3′ fragment) containing overlapping ends using the following specific primers:

**Table 1:** Primer pairs used in the construction of *ABCD2* promoter fragments.

Constructs	Positions	Primer Sequences#	
−1300 luc	−1302/−1	Forward	5′-*GTACTCGAG* GAAACCTGCAAAAGACAA-3′
		Reverse	5′-*GGACAAGCTT* TTTCCCAGTTACCCAAAC-3′
−800 luc	−800/−1	Forward	5′-*GAACTCGAG* TCTTTTCTGATCCGTTTC-3′
		Reverse	5′-*GGACAAGCTT* TTTCCCAGTTACCCAAAC-3′
−500 luc	−499/−1	Forward	5′-*GAACTCGAG* ACTGAAATCTTACCGAAG-3′
		Reverse	5′-*GGACAAGCTT* TTTCCCAGTTACCCAAAC-3′
−1300/−500 luc	−1302/−500	Forward	5′-*GTACTCGAG* GAAACCTGCAAAAGACAA-3′
		Reverse	5′-*GGACAAGCTT* AGATACCGCAAACAGAAG-3′
−360 luc	−360/−1	Forward	5′-*GAACTCGAG* CTCTCTGAACTCCTGTTT-3′
		Reverse	5′-*GGACAAGCTT* TTTCCCAGTTACCCAAAC-3′
−260 luc	−260/−1	Forward	5′-*GAACTCGAG* CTCACAGCCAATGAGGGG-3′
		Reverse	5′-*GGACAAGCTT* TTTCCCAGTTACCCAAAC-3′
−160 luc	−160/−1	Forward	5′-*GAACTCGAG*ATCTGTCACAGCAGAACA-3′
		Reverse	5′-*GGACAAGCTT* TTTCCCAGTTACCCAAAC-3′

# NOTE: Underlined italic letters represent irrelevant sequences and restriction enzyme sites (XhoI and HindIII) used for cloning.

mTBE1-5′primer, 5- GATGTAATTCG**CCCTGCT**CTCTCCTCA -3 and

mTBE1-3′ primer, 5- TGAGGAGAG**AGCAGGG**CGAATTACATC -3;

mTBE2-5′ primer, 5- TTCAAGG**TTAGAAG**GGAAGCTGCGAGG -3 and

mTBE2-3′ primer, 5- CCTCGCAGCTTCC**CTTCTAA**CCTTGAA -3. Putative LEF-1/TCF binding sites are shown in bold, and mutated sequences are underlined. After two rounds of PCR, each full-length construct with the desired mutation (800 bp DNA fragment) was amplified with primers for 800-luc (Table 1), and subcloned into the pGL3-basic vector. A construct with mutations in both TBEs (Dm TBE) was generated by further mutation of TBE2 from a TBE1-mutated plasmid. The sequences of all reporter constructs were verified by DNA sequencing (SolGent, Korea).

### Transfection and Luciferase Reporter Assays

For reporter assays, HepG2 cells were seeded in a 24-well culture plate and transiently transfected with 0.3 µg reporter plasmids and 50 ng of pRL-SV40 plasmid (Promega) containing the *Renilla* luciferase gene under the control of the Simian virus 40 promoter using Fugene6 transfection reagent (Roche). The total amount of transfected DNA was kept constant by adding an empty vector. Fibroblasts isolated from an X-ALD patient were electroporated with 0.5 µg reporter plasmids and 0.2 µg of pRL-SV40 plasmid using a microporator transfection system (Neon™, Invitrogen). Cells were pulsed twice with a voltage of 1,100 (V) for 30 ms. After the two pulses, cells were seeded in a 12-well culture plate. At 36 h after transfection (or electroporation), cells were harvested and luciferase activities were determined by measuring luminescence activity. Data were normalized by *Renilla* luciferase activity. For overexpression of *β*-catenin and TCF-4, cells were transfected (or electroporated) with each plasmid using Fugene6 (for HepG2 and Huh7) or a microporator (for X-ALD fibroblasts). Forty-eight hours after transfection, cells were harvested for luciferase activity measurement, western blot analysis, and immunofluorescence staining.

### RNAi

Scrambled, *β-catenin* and *ABCD2* siRNA were purchased from Genolution Pharmaceuticals (Korea). HepG2 or X-ALD fibroblasts were transfected with scrambled siRNA (5-ACGUGACACGUUCGGAGAA-3), *β-catenin* siRNA (5-CUGGGACCUUGCAUAACCU-3) or *ABCD2* siRNA (5-GAUUAUGUCUUCAUACAAA-3 and 5-GCAUGAUAAAGGUUAUACA-3) using Lipofectamine™ RNAiMAX (Invitrogen). Three to four days after transfection, cells were harvested to measure mRNA or protein levels, luciferase activities, and VLCFA levels.

### Chromatin Immunoprecipitation (ChIP) Assay

ChIP assay was performed as described previously [30]. In brief, cells were grown to 80% confluency on 100-mm culture dishes, and then the proteins in cells were cross-linked to DNA by adding formaldehyde for 10 min at RT. Cells were then washed and sonicated to shear chromatin DNA to less than 1000 bp in length. Sheared chromatin was precleared by adding protein G-agarose/salmon sperm beads (Millipore) and then incubated with either 1 µg anti-rabbit IgG or anti-*β*-catenin antibodies overnight at 4°C, then for another 2 h at 4°C with protein G-agarose/salmon sperm beads. Following washes, the protein/DNA cross-links were reversed by heating at 65°C overnight, and the DNA fragments eluted. Eluted DNA fragments were used for PCR analysis using the following human *ABCD2* promoter-specific primers:

forward, 5-GTTTTGTTCGCCAGCAGATGGCCTGAT-3;

reverse, 5-CCGCTGCATCTACCGGGAATGATTCTC-3. Amplified DNA fragments were visualized by agarose gel electrophoresis.

### RNA Isolation and Real-time PCR

Total RNAs were isolated from cells using TRIzol reagent (Invitrogen). The cDNAs were synthesized usingthe iScript™ cDNA synthesis kit (Bio-Rad) from 1 µg of total RNA. Expression of *β-catenin*, *ABCD2*, and *GAPDH* was determined by real-time PCR using SYBR® Premix Ex Taq (Takara) on a CFX Connect™ real-time PCR system (Bio-Rad) according to the manufacturer’s instructions. Real-time PCR was performed using the following specific primer sequences: *β-catenin*-f, 5-ATGTCCAGCGTTTGGCTGAA-3 and.

*β-catenin* -r, 5-TGGTCCTCGTCATTTAGCAGTT-3;

*ABCD1*-f, 5-GGAGCTGGTGGCAGAGGA-3 and

*ABCD1*-r, 5-ACAGCCACCATGAGCAGG-3;

*ABCD2*-f, 5-GATAACTGGTCCCAATGGTTG-3 and

*ABCD2*-r, 5-TCCCGAAGACTTCCAAGAGA-3;

*GAPDH* -f, 5-CCCCTCAAGGGCATCCTGGGCTA-3 and

*GAPDH* -r, 5-GAGGTCCACCACCCTGTTGCTGTA-3.

### Western Blot Analysis

Total cells lysates were prepared using cell lysis buffer containing protease inhibitor cocktail (Roche) and sodium orthovanadate (Sigma) for 20 min on ice as described previously [31]. The Bradford assay (Bio-Rad) was used to determine protein concentration. Equal amounts of protein were subjected to 10% SDS-PAGE and transferred to a PVDF membrane (Millipore). The membrane was blocked and then incubated for 2 h at RT with one of the following antibodies: anti-β-catenin antibody (Cell Signaling Technology), anti-β-actin and anti-Flag antibody (Sigma), or anti-Myc antibody (Santa Cruz Biotechnology). Following three washes, the membranes were incubated with horseradish peroxidase-conjugated secondary antibodies (Jackson Immuno Research Laboratories) for 1 h at RT. Antibody-labeled proteins were visualized using ECL solution (Amersham Biosciences).

### Confocal Microscopy

X-ALD fibroblasts were electroporated with ABCD2-GFP expression plasmid, and cells were seeded on cover glass. Two days after electroporation, cells were washed in phosphate-buffered saline (PBS) and fixed in 4% paraformaldehyde and 0.1% Triton X-100 for 20 min at RT. Cells were further incubated in 5% bovine serum albumin (BSA) for 20 min at RT, and then incubated with anti-PMP70 polyclonal antibody (Millipore) for 2 h at 37°C. After three washes in PBS, cells were incubated with Alexa Fluor 594-conjugated donkey anti-rabbit IgG (Invitrogen) for 1 h at 37°C. After two washes with 0.1% Triton X-100 in PBS and three washes in PBS, cells were analyzed using a confocal microscopy system (Olympus).

### VLCFA Analysis

X-ALD fibroblasts were harvested by trypsinization, and the cell pellet (2×10^5^ cells) was dissolved in PBS. VLCFA analysis was performed by Seoul Clinical Laboratories (Korea) as described previously [32]. VLCFAs were determined by methyl ester formation [33]. In brief, heptacosanoic acid (C27∶0) was added as the internal standard to each sample. Then methylene chloride in methanol and acetyl chloride were added and the samples were heated for 1 h at 75°C to allow the formation of methyl esters. After cooling, potassium carbonate solution was added to quench the reaction by neutralization. The resulting fatty acid methyl esters were extracted with hexane solution, followed by extraction with acetonitrile to remove polar compounds. The hexane layer was taken and evaporated to dryness under a gentle stream of nitrogen. The dry residue was reconstituted in hexane for gas chromatography analysis.

### Statistical Analysis

The data are presented as means± SEMs. Student’s *t*-test was used for statistical analysis. *p*<0.05 was considered statistically significant.

## Results

### Identification of LEF-1/TCF Binding Sites within the Promoter of the Human *ABCD2* Gene

**Figure 1:**
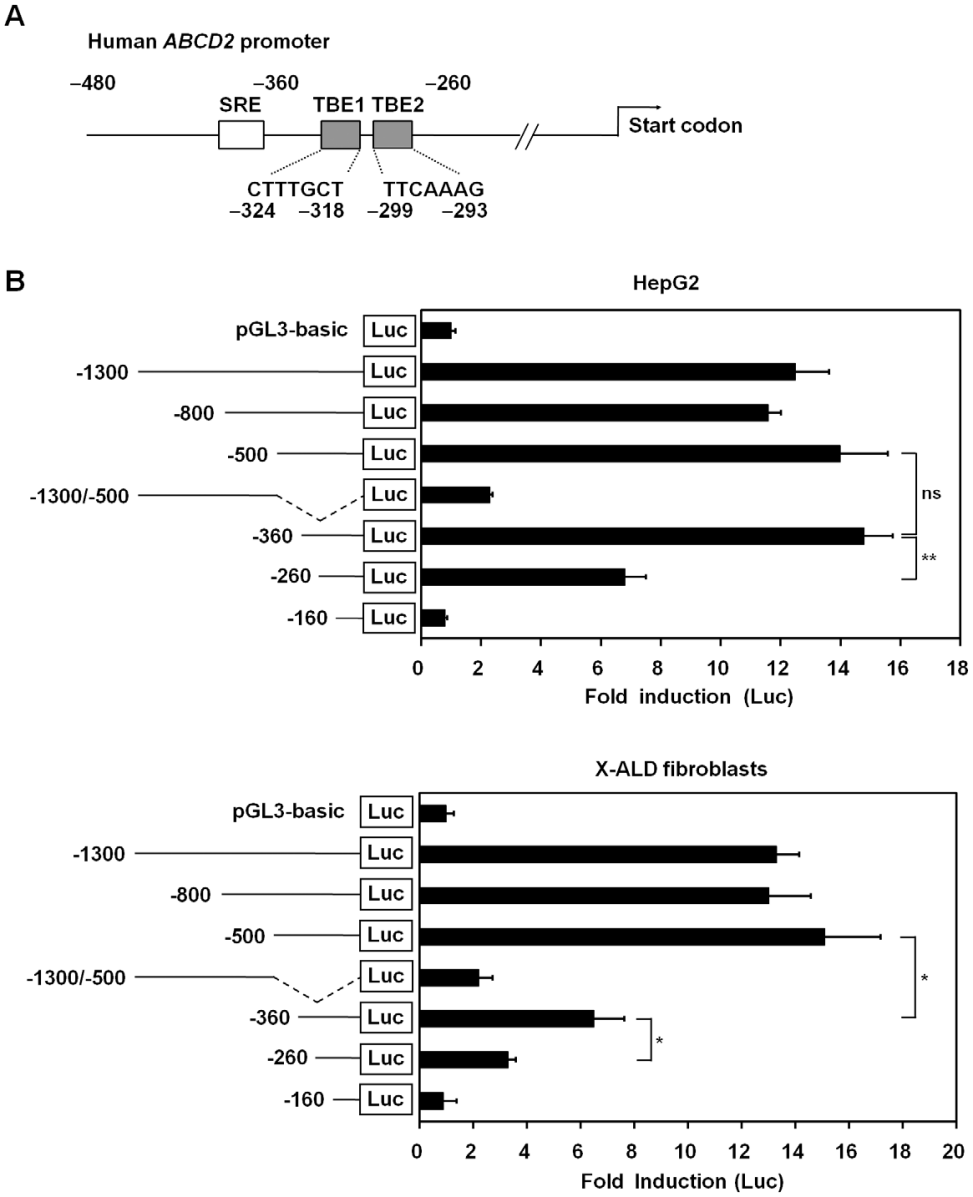
Analysis of luciferase reporter constructs to determine the minimal DNA sequence required for human *ABCD2* promoter activity. (A) Schematic representation of the human *ABCD2* promoter showing the two putative LEF-1/TCF binding sites identified with Genomatix software (MatInspector™). The two putative TCF binding sites (TBE1 and TBE2) are located at nucleotide positions −324 to −318 and −299 to −293 relative to the ATG translational start site in the upstream sequence of *ABCD2* promoter. (B) Each reporter construct was transfected into HepG2 cells using Fugene6 (upper panel), whereas these constructs were electroporated into fibroblast cells from an X-ALD patient with a microporator (lower panel). Thirty-six hours after transfection, cells were harvested, and luciferase activities were determined and normalized by *Renilla* luciferase activity. Data represent the mean (±SEM) of triplicate experiments and each experiment was repeated three times. *p<0.05; **p<0.01; ns, not significant.

To investigate transcription factors that could potentially regulate *ABCD2*, we first analyzed transcription factor binding elements with a proximal region of the promoter of this gene. The upstream sequence (−1300 bp) of the *ABCD2* promoter subcloned in this study was identical to that of reported sequence on human chromosome 12 (NCBI, Gene ID: 225) (data not shown). By in silico analysis with Genomatix software (MatInspector™), we identified the two putative LEF-1/TCF binding elements (TBEs) located at nucleotide positions −324 to −318 (TBE1) and −299 to −293 (TBE2) within the upstream sequence of the *ABCD2* promoter (Fig. 1A). To investigate the involvement of *β*-catenin/TCF-4 on transcriptional activation of the *ABCD2* gene, we measured promoter activity from various lengths of the *ABCD2* promoter in HepG2 cells expressing constitutively active *β*-catenin protein [34]. We found that the −1300 bp, −800 bp, and −500 bp promoter fragments showed full transcriptional activity compared to the pGL3 basic group (negative control group) (Fig. 1B, upper panel). However, a fragment containing −1300/−500 bp of the *ABCD2* promoter showed only a 2.3-fold increase in activity compared to the negative control group (Fig. 1B, upper panel). Similar results were obtained for the X-ALD fibroblasts isolated from a patient (Fig. 1B, lower panel). These data indicate that the region 500 bp upstream of the *ABCD2* promoter is an important region for transcriptional activity in both cell types. Interestingly, the −360 bp fragment, which does not contain a sterol regulatory element (SRE) showed full transcriptional activity in HepG2 cells (Fig. 1B, upper panel). In addition, the promoter activity of a −260 bp fragment lacking the two TBEs sequence was less than half that shown by the −500 bp or −360 bp fragments (Fig. 1B, upper panel). In contrast, in the X-ALD fibroblasts, the promoter activity of the −360 bp fragment was half of that obtained from the −500 bp fragment, while the promoter activity of the −260 bp fragment was significantly lower than that of the −360 bp fragment (Fig. 1B, lower panel). These results suggest the existence of a potential positive regulatory element(s) within the −360/−260 region of the *ABCD2* promoter in both cell types.

### Activation of *ABCD2* Promoter by *β*-catenin and TCF-4

**Figure 2:**
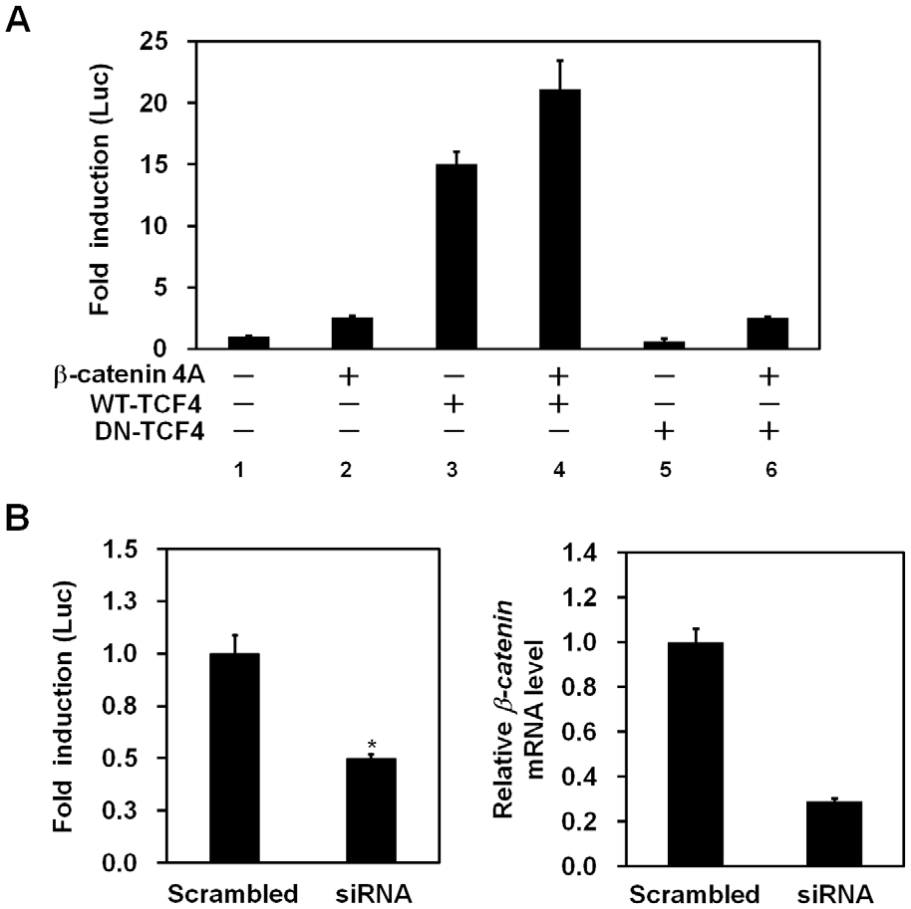
*β*-catenin and TCF-4 increase the transcriptional activity of *ABCD2*. (A) HepG2 cells were cotransfected with *ABCD2* promoter reporter (800-Luc) plasmids together with the indicated expression plasmids. At 36 h after transfection, cells were harvested, and luciferase activities were determined. The amount of DNA in each transfection was kept constant by adding an appropriate amount of pcDNA3 empty vector. (B) HepG2 cells were transfected with either scrambled or *β-catenin* siRNA and were then further transfected with 800-Luc 24 h later. Luciferase activity was determined 48 h later (left panel). The efficacy of silencing was verified by real-time PCR (right panel). Data represent the mean (± SEM) of triplicate experiments and each experiment was repeated three times. *p<0.05.

To further investigate transcriptional regulation of *ABCD2*, cells were transfected with the 800-Luc reporter plasmid alone or combinations with *β*-catenin active mutant (4A), wild-type TCF-4, dominant negative (DN)-TCF-4 expression plasmids. As shown in Fig. 2A, expression of the active *β*-catenin mutant protein resulted in only 2.6-fold activation of the *ABCD2* promoter (Fig. 2A, *lane 2*) due to the presence of an endogenous active mutant form of *β*-catenin in HepG2 cells [34]. Interestingly, the promoter activity of the *ABCD2* gene was 15-fold greater in cells expressing wild type TCF-4 protein than cells expressing empty vector (Fig. 2A, *lane 3*). In contrast, promoter activity was slightly decreased in cells that expressed DN-TCF-4 protein (Fig. 2A, *lane 5*). These results strongly suggest that *β*-catenin and TCF-4 interact to activate transcription of the *ABCD2* gene. Consistent with these results, we found that cotransfection of *β*-catenin and wild-type TCF-4 expression plasmids resulted in a 21.1-fold induction in promoter activity (Fig. 2A, *lane 4*), whereas cotransfection of *β*-catenin and DN-TCF-4 plasmids resulted in only a 2.5-fold induction in promoter activity (Fig. 2A, *lane 6*).

To determine the role of *β*-catenin in *ABCD2* promoter transactivation in HepG2 cells, endogenous *β*-catenin expression was knocked-down using specific siRNAs. Real-time PCR analysis revealed that the expressions of both wild-type (WT) and active mutant forms (Mut) of *β*-catenin in HepG2 cells were efficiently reduced by siRNA transfection (Fig. 2B, right panel). Subsequently, we showed that the promoter activity of the *ABCD2* gene was decreased by *β*-catenin silencing (Fig. 2B, left panel). These data indicate that both *β*-catenin and TCF-4 are required for the transcriptional activation of *ABCD2*.

### Contribution of the Two TBE Sites within the *ABCD2* Promoter to *β*-catenin/TCF-4 Mediated Transcriptional Activation

**Figure 3:**
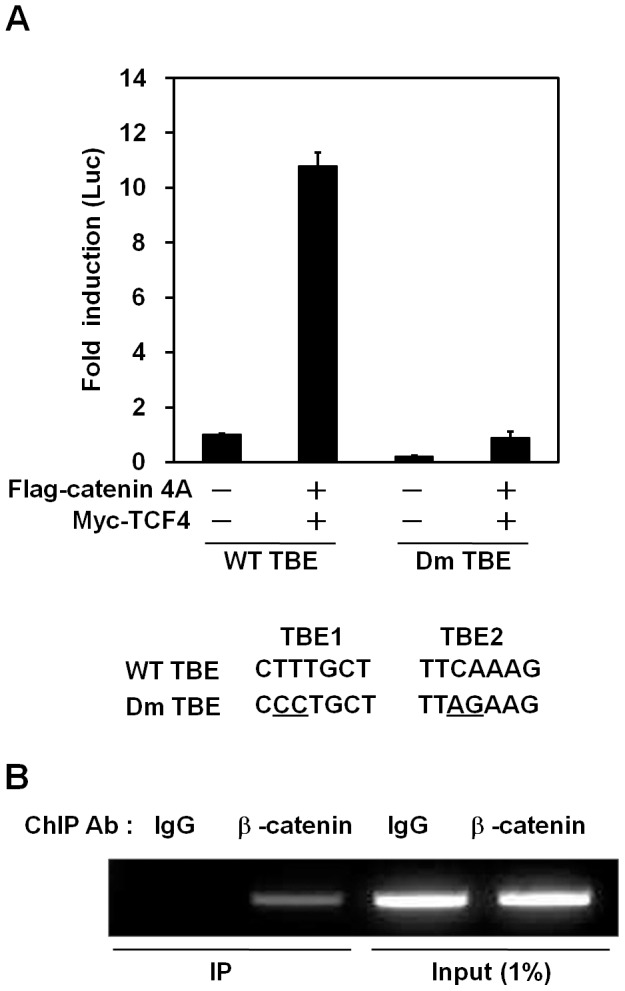
Contribution of the two TBE sites within the *ABCD2* promoter to *β*-catenin/TCF-4 mediated transcriptional activation. (A) HepG2 cells were cotransfected with the indicated expression plasmids along with either wild-type (WT) 800-Luc reporter plasmid containing wild-type (WT) sequences or double mutant (Dm) 800-Luc reporter plasmid containing mutations in the two TBE sites of the *ABCD2* promoter. Mutated sequences are underlined (lower panel). At 36 h after transfection, cells were harvested, and luciferase activities were determined. The amount of DNA in each transfection was kept constant by adding an appropriate amount of pcDNA3 empty vector. Data represent the mean (± SEM) of triplicate experiments. (B) A ChIP assay was performed by immunoprecipitation of DNA/protein complexes from HepG2 cells with an anti-*β*-catenin antibody. The immunoprecipitated DNA was amplified with primers specific for the *ABCD2* promoter. Purified rabbit IgG (IgG) was used as a negative control. Input DNA (before immunoprecipitation) was amplified as a positive control for the PCR. The amplified DNA was analyzed by agarose gel electrophoresis.

To determine whether the two putative TBEs identified in this study are required for *β*-catenin*/*TCF-4-mediated transcriptional activation, we introduced mutations into the TBEs and performed luciferase assays. To destroy the binding sequence of LEF-1/TCF, we mutated two nucleotides within each TBE motif (Fig. 3A, lower panel). First, we investigated the effect of mutations in TBE sites on promoter activation in Huh7 cells. Mutation of either of the TBE sites significantly decreased transcriptional activation by *β*-catenin/TCF-4 (Fig. S1). However, the greatest reduction was observed when both TBE sites were mutated (Fig. S1), hence we used the construct with mutations of both TBE sites for further experiments in HepG2 cells. As expected, we found that mutation of both TBE sites strongly reduced transcriptional activation by *β*-catenin/TCF-4 as well as basal levels of transcription (Fig. 3A). In the absence of ectopic expression of *β*-catenin/TCF-4, luciferase activity was decreased 5-fold by of the TBE mutations. Moreover, ectopic expression of *β*-catenin/TCF resulted in a 10.8-fold induction in *ABCD2* promoter activity when wild-type TBEs sequences were present, compared to a 4.5-fold induction when the TBEs were both mutated (Fig. 3A). These results indicate that *β*-catenin/TCF-4-mediated transcriptional activation of *ABCD2* can be blocked by mutating the TCF-4 binding elements in the *ABCD2* promoter region. Similar results were obtained in the X-ALD fibroblasts (Fig. S2).

To determine if the *ABCD2* promoter is a direct target of the *β*-catenin/TCF-4 complex, we performed a chromatin immunoprecipitation (ChIP) assay. We used either normal rabbit IgG or *β*-catenin antibodies for ChIP, and amplified the DNA using specific primers for the *ABCD2* promoter containing the two TBEs. As shown in Fig. 3B, the *ABCD2* promoter was immunoprecipitated by the *β*-catenin antibody, but was not immunoprecipitated by the normal IgG antibody. These data indicate that a transcription factor complex containing *β*-catenin binds directly to the *ABCD2* promoter.

### Upregulation of *ABCD2* Gene Expression via *β*-catenin and TCF-4

**Figure 4:**
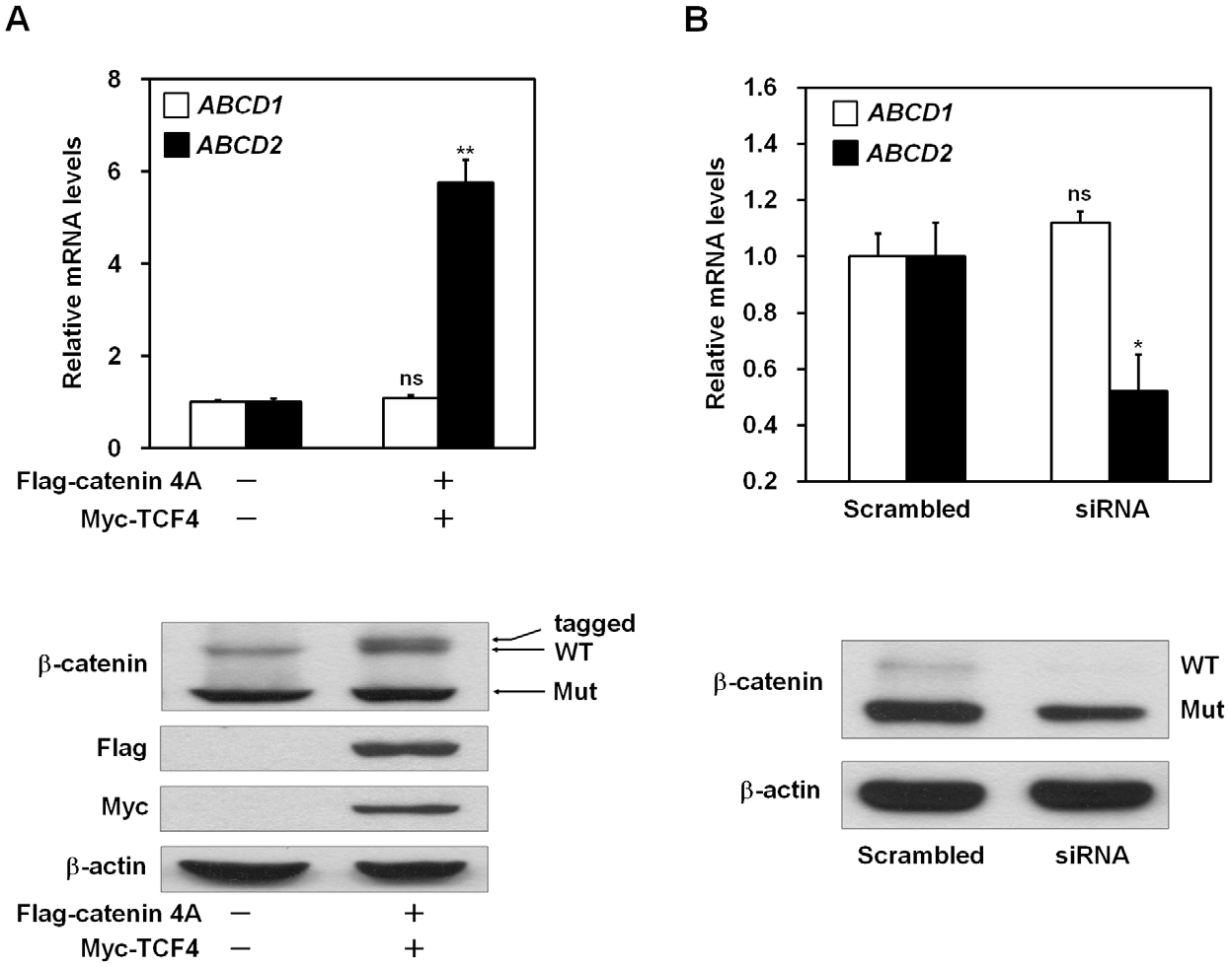
*β*-catenin and TCF-4 up-regulate mRNA levels of *ABCD2*. HepG2 cells were cotransfected with Flag-tagged constitutively active *β*-catenin (Flag-catenin 4A) and Myc-tagged TCF-4 expression plasmids. (A) At 48 h after transfection, mRNA was isolated from transfected cells. The levels of *ABCD1* and *ABCD2* mRNA were measured by real-time PCR and were normalized to *GAPDH* expression (upper panel). Data represent the mean (± SEM) of triplicate experiments. Total cell lysates harvested from transfected cells were immunoblotted with the indicated antibodies (lower panel). Protein expression of *β*-actin was used as a loading control. **p<0.001; ns, not significant; control vector transfected cells *versus β*-catenin and TCF-4 transfected cells. (B) HepG2 cells were transfected with either scrambled or *β-catenin* siRNA. At 3 days after transfection, mRNA was isolated from transfected cells. The levels of *ABCD1* and *ABCD2* mRNA were measured by real-time PCR and normalized to *GAPDH* expression (upper panel). *p<0.05; ns, not significant; scrambled transfected cells *versus β-catenin* siRNA transfected cells. Efficacy of silencing was verified by western blotting with anti-*β*-catenin antibody (lower panel). Protein expression of *β*-actin was used as a loading control.

We examined whether mRNA level of *ABCD2* gene was increased by ectopic expression of active mutant *β*-catenin (4A) and wild-type TCF-4 in HepG2 cells. mRNA expression of *ABCD2* was compared between control vector-transfected or *β*-catenin and TCF-4-transfected cells after total RNA extraction. As shown in Fig. 4A, *ABCD2* expression was elevated by ectopic expression of *β*-catenin and TCF-4. In contrast, *ABCD1* expression was unaffected by these proteins (Fig. 4A). We also confirmed the increased expression of transfected *β*-catenin and TCF-4 proteins by western blot analysis (Fig. 4A, lower panel). Unfortunately, we were unable to detect an increased protein level of ABCD2 as a result of commercially available antibodies not working against the ABCD2 protein. Conversely, transcript expression of *ABCD2* was significantly decreased by siRNA-mediated *β-catenin* knockdown in HepG2 cells (Fig. 4B). However, *ABCD1* expression was not decreased by *β-catenin* knockdown (Fig. 4B). We verified the knockdown of *β*-catenin by western blotting (Fig. 4B, lower panel) using a specific antibody against *β*-catenin.

### Restoration of VLCFA Levels in the Fibroblasts Derived from an X-ALD Patient by Ectopic Expression of ABCD2-GFP

**Figure 5:**
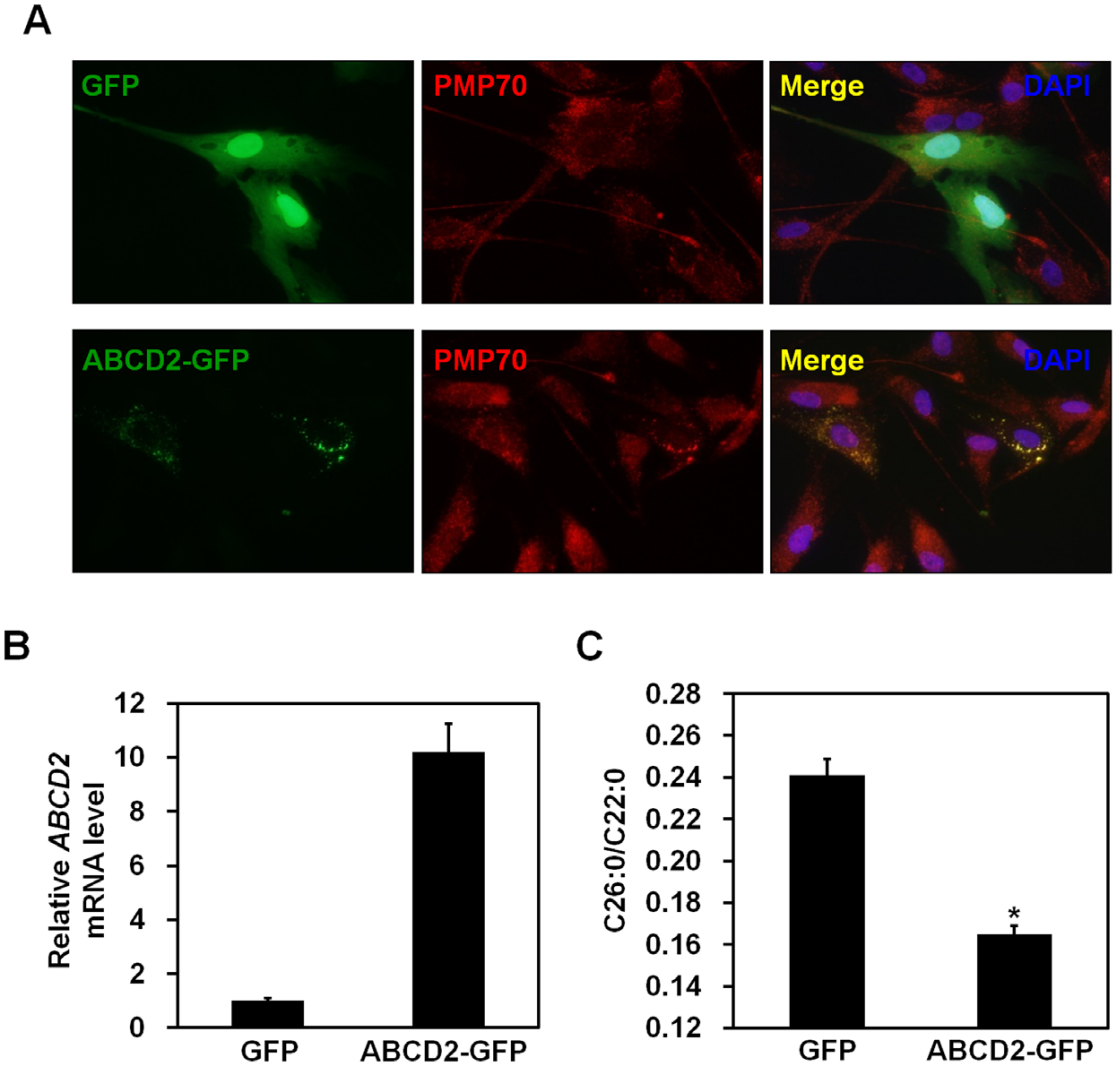
Levels of VLCFAs in fibroblasts from an X-ALD patient were restored by ectopic expression of ABCD2-GFP. Primary fibroblasts isolated from an X-ALD patient were electroporated with either GFP or ABCD2-GFP expressing plasmid using a microporator. (A) At 48 h after electroporation, cells were fixed and incubated with anti-PMP70 antibody for 2 h. After washing with PBS, cells were incubated with Alexa Fluor 594-conjugated donkey anti-rabbit IgG for 1 h. Immunofluorescence staining was analyzed using a confocal microscopy system. Cells were counterstained with 4′,6-diamidino-2-phenylindole (DAPI) to label nuclei. (B) At 48 h after electroporation, mRNA was isolated from cells. The levels of ectopically expressed *ABCD2* were measured by real-time PCR and normalized to *GAPDH* expression. Data represent the mean (± SEM) of triplicate experiments. (C) At 4 days after electroporation, cells were harvested and the levels of VLCFA were measured. The values are expressed as the ratio of C26∶0 to C22∶0. Data represent the mean (± SEM). *p<0.01; GFP-expressing cells *versus* ABCD2-GFP-expressing cells.

Functional replacement of the ABCD1 protein (ALDP) by the ABCD2 protein (ALDRP) has been shown to reduce VLCFA accumulation in fibroblast cells isolated from X-ALD patients [10], [12]. To confirm this finding in our cell system, either GFP or ABCD2-GFP expression vectors were electroporated into fibroblast cells isolated from an X-ALD patient. A punctuate spot pattern of GFP signals from the ABCD2-GFP protein was detected outside of the nucleus, and this pattern merged perfectly with fluorescent signals from PMP70, which is a peroxisomal marker protein (Fig. 5A). These results indicate that the ABCD2-GFP fusion protein is able to correctly target the peroxisomal membrane.

Since C26∶0/C22∶0 ratio is being used widely for diagnosing X-ALD [33], we analyzed the ratio of C26∶0/C22∶0 to determine if the ABCD2 protein had a compensatory effect. We found that ectopic expression of ABCD2-GFP by electroporation resulted in a 10-fold induction in ABCD2-expression in fibroblasts relative to the negative control (Fig. 5B). Interestingly, we observed that ectopic expression of GFP-tagged ABCD2 resulted in a decrease in the ratio of C26∶0/C22∶0, indicating that accumulated VLCFA levels were reduced in the fibroblast cells from an X-ALD patient by ectopic ABCD2 (Fig. 5C). These results in our cell system are consistent with previous studies that reported that ABCD2 is functionally redundant with ABCD1 [10]–[12].

### *β*-catenin and TCF-4 Regulate the Levels of VLCFAs via Induction of *ABCD2* Gene

**Figure 6:**
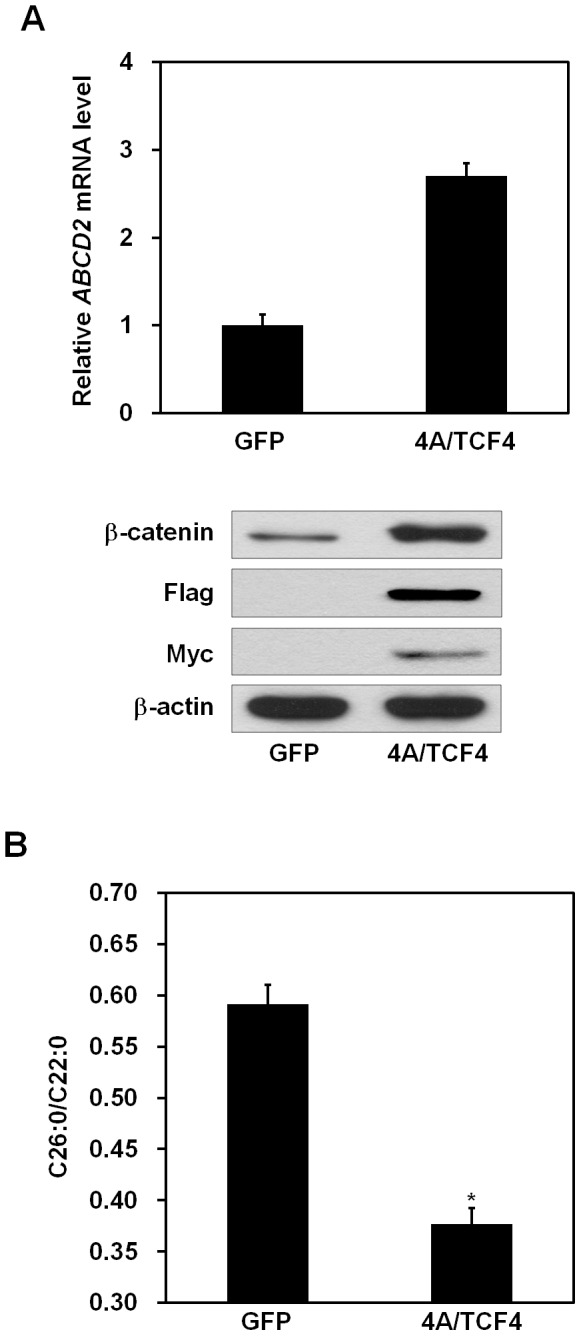
*β*-catenin and TCF-4 reduce the levels of VLCFAs by affecting *ABCD2* gene induction. Primary fibroblasts isolated from an X-ALD patient were electroporated with Flag-tagged constitutively active *β*-catenin (4A active mutant) and Myc-tagged TCF-4 expression plasmids using a microporator. (A) At 48 h after electroporation, mRNA was isolated from cells. The levels of *ABCD2* mRNA were measured by real-time PCR and normalized to *GAPDH* expression (upper panel). Data represent the mean (± SEM) of triplicate experiments. At 48 h after electroporation, total cell lysates harvested from transfected cells were immunoblotted with the indicated antibodies (lower panel). Protein expression of *β*-actin was used as a loading control. (B) Four days after electroporation, cells were harvested and the levels of VLCFA were measured. Values are expressed as the ratio of C26∶0 to C22∶0. Data represent the mean (± SEM). *p<0.001; GFP-expressing cells *versus β*-catenin and TCF-4-expressing cells.

**Figure 7:**
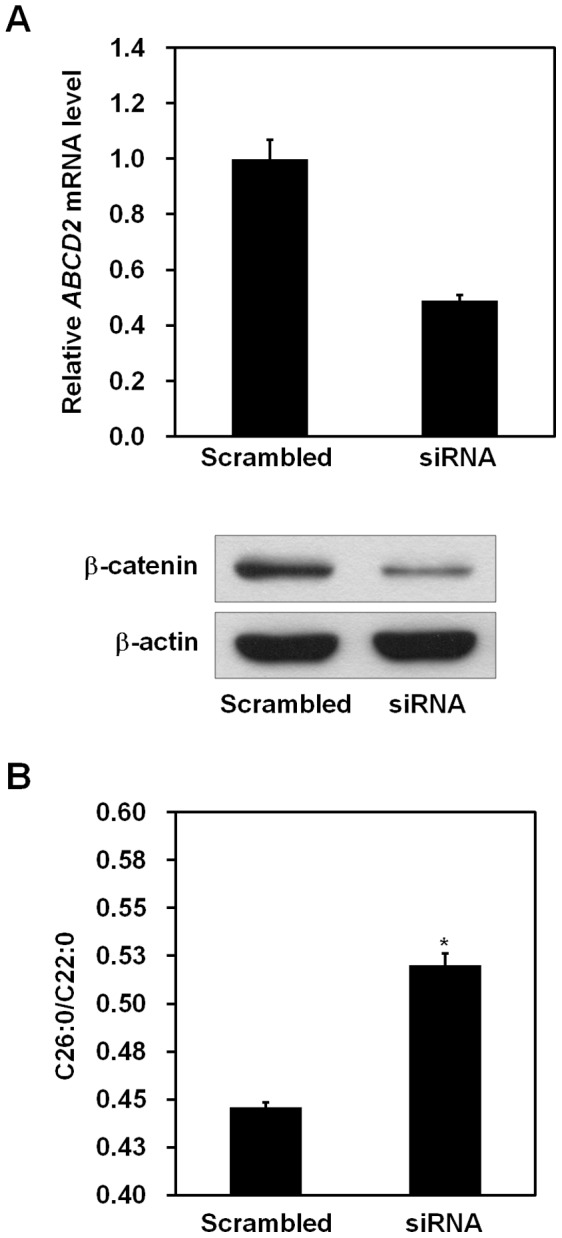
Silencing of *β-catenin* in X-ALD fibroblasts increased VLCFA levels. Primary fibroblast cells isolated from an X-ALD patient were transfected with either scrambled or *β-catenin* siRNA. (A) At 3 days after transfection, mRNA was isolated from transfected cells. The levels of *ABCD2* mRNA were measured by real-time PCR and were normalized to *GAPDH* expression (upper panel). Data represent the mean (± SEM) of triplicate experiments. At 3 days after transfection, the efficacy of silencing was verified by western blotting with anti-*β*-catenin antibody (lower panel). Protein expression of *β*-actin was used as a loading control. (B) Four days after transfection, cells were harvested and the levels of VLCFA were measured. The values are expressed as the ratio of C26∶0 to C22∶0. Data represent the mean (± SEM). *p<0.001; scrambled transfected cells *versus β-catenin* siRNA-transfected cells.

**Figure 8:**
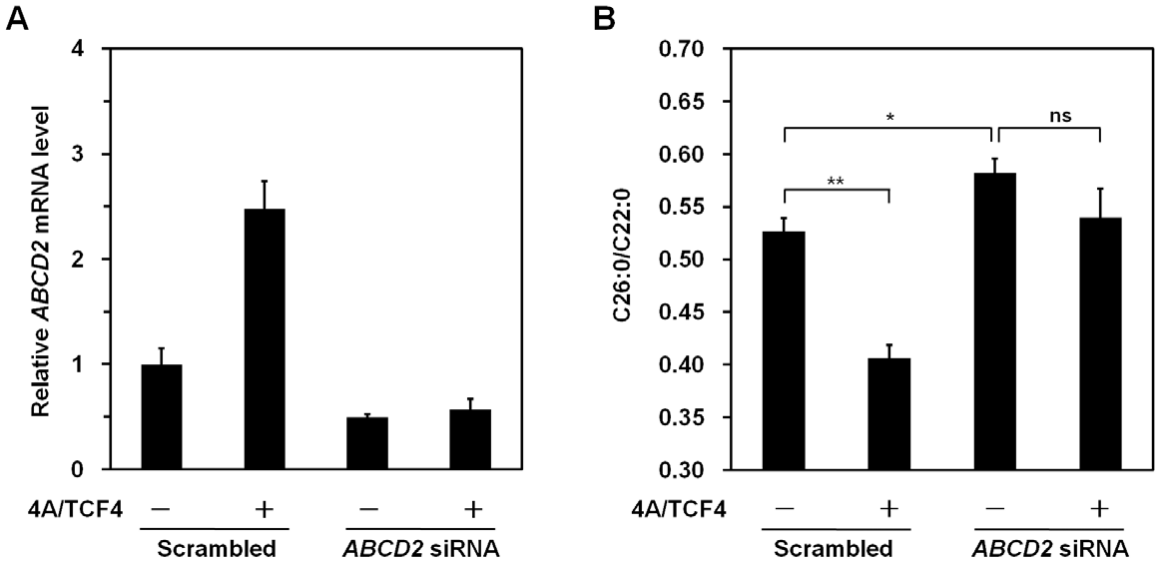
*β*-catenin and TCF-4 regulate the levels of VLCFAs through *ABCD2* gene induction. X-ALD fibroblast cells were transfected with either scrambled or *ABCD2* siRNA. Two days after transfection, cells were electroporated with Flag-tagged constitutively active *β-*catenin (4A active mutant) and Myc-tagged TCF-4 expression plasmids using a microporator. (A) Two days after electroporation, mRNA was isolated from cells. The levels of *ABCD2* mRNA were measured by real-time PCR and normalized to *GAPDH* expression. Data represent the mean (± SEM) of triplicate experiments. (B) Four days after electroporation, cells were harvested and the levels of VLCFA were measured. Values are expressed as the ratio of C26∶0 to C22∶0. Data represent the mean (± SEM). *p<0.05; **p<0.01; ns, not significant.

We next asked whether VLCFA levels decreased in response to *β*-catenin and TCF-4-induced *ABCD2* induction in X-ALD fibroblasts. X-ALD fibroblasts were electroporated with either GFP or active *β*-catenin and TCF-4 expression vectors. We showed that mRNA level of the *ABCD2* gene was increased by overexpression of *β*-catenin and TCF-4 using real-time PCR analysis (Fig. 6A, upper panel). Interestingly, we found that the ratio of C26∶0/C22∶0 was significantly decreased by overexpression of these proteins (Fig. 6B). Conversely, mRNA levels of *ABCD2* decreased when *β-catenin* was silenced (Fig. 7A). In addition, silencing of *β-catenin* resulted in a significant increase in VLCFA levels (Fig. 7B). We further investigated whether *β*-catenin/TCF-4-dependent change of C26∶0/C22∶0 is mediated by induction of *ABCD2*. For this purpose, X-ALD fibroblasts were transfected with *ABCD2* siRNA. We observed that *β*-catenin/TCF-4-dependent change of C26∶0/C22∶0 was abolished by *ABCD2* knockdown in X-ALD fibroblasts (Fig. 8). These data indicate that *β*-catenin/TCF-4 are able to regulate the levels of VLCFAs through *ABCD2* gene induction.

## Discussion

X-ALD, caused by mutations of the *ABCD1* gene, is associated with the accumulation of VLCFAs in the plasma and tissues of patients [35]. At present, no therapeutic drug for X-ALD is available; although Lorenzo’s oil reduces the levels of plasma VLCFA with little or no effect on the disease progression [36]. The cholesterol-lowing drug, lovastatin, was suggested as a potential therapeutic drug for X-ALD [37], [38], but did not decrease VLCFA levels in a placebo-controlled clinical trial [39].

Induction of *ABCD2* may be a promising treatment option for X-ALD because expression of this protein can decrease VCLFA levels in fibroblasts. However, little is known about transcriptional activation of this gene or the mechanism of action of drugs that have been reported to activate this gene, although a binding site for SREBP1 (SRE at position −401/−391) in the *ABCD2* promoter has been reported [25]. We therefore investigated transcription factors involved in the regulation of *ABCD2* gene expression.

By in silico analysis using Genomatix software (MatInspector™), we found two putative LEF-1/TCF binding elements (TBEs) (Fig. 1A) in the promoter region of *ABCD2* in addition to the GC and CCAAT boxes reported in earlier studies [40], [41]. The TBE1 sequence (CTTTGCT) is one nucleotide different from the TCF-4 binding sequence (CTTTG(A/T)(A/T)) [42], whereas the TBE2 sequence (TTCAAAG) is an inverted perfect match of the consensus sequence (Fig. 1A). To investigate if these sites are involved in *ABCD2* gene transcription, we performed luciferase reporter assays using various lengths of the *ABCD2* promoter. Because the promoter of the *ABCD2* gene has putative TBEs, we chose HepG2 cells that contain active *β*-catenin protein to investigate transcriptional activation. In both HepG2 cells and fibroblasts from an X-ALD patient, the −260 promoter fragment showed minimal activity, while the activity of the −360 fragment differed according to cellular context (Fig. 1). We further demonstrated that TBEs are involved in transcriptional regulation of *ABCD2* by performing a mutagenesis study (Fig. 3A). Interestingly, we found that the −260 fragment still had promoter activity in both cell types even when the TBE sequences were deleted (Fig. 1A and 1B), indicating the existence of another response element within the promoter region.

Next, we showed that the promoter activity of *ABCD2* was elevated by ectopic expression of *β*-catenin and TCF-4 (Fig. 2A, *lane 4*). We confirmed this finding in Huh7 cells that do not express active *β*-catenin [43] (Fig. S3) and in CCALD fibroblast cells (data not shown). Site-directed mutagenesis results provided direct evidence that the TBEs within the *ABCD2* promoter are functionally involved in *ABCD2* transcriptional regulation (Fig. 3A). Through ChIP assays, we showed that *β*-catenin containing complexes bind directly to the promoter region of the *ABCD2* gene (Fig. 3B). In addition, we demonstrated that gene expression of *ABCD2* is upregulated by overexpression of *β*-catenin/TCF-4 (Figs. 4A and 6A) while downregulation occurs by silencing of *β-catenin* (Figs. 4B and 7A) in both HepG2 cells and X-ALD fibroblasts. Moreover, we showed that *β*-catenin/TCF-4 reduced the ratio of C26∶0/C22∶0 (Fig. 6B) as well as the levels of C24∶0 and C26∶0 (data not shown) in the fibroblasts from an X-ALD patient. Finally, we showed that this effect was reversed by *β-catenin* silencing (Fig. 7B).

Transcription factors such as SREBPs [25], [26], triiodothyronine (*T*_3_)-liganded thyroid hormone receptor α (TRα)/SREBP-1 [44], peroxisome proliferator-activated receptor α (PPARα) [42], and the retinoic X receptor (RXR)/*cis*-retinoic acid [45] have been reported to be regulators of the *ABCD2* gene. However, we found that *β*-catenin and TCF-4 not only increased transcription of the *ABCD2* gene, but also decreased levels of VLCFAs according to the level of *ABCD2* induction. We therefore speculate that induction of *ABCD2* by *β*-catenin and TCF-4 may decrease levels of VLCFAs.

In this study, we demonstrated direct regulation of *ABCD2* gene expression by *β*-catenin and TCF-4, which are members of the canonical Wnt signaling pathway. These findings may provide a new strategy for developing drugs to treat X-ALD.

## Supporting Information

Figure S1 **Mutation of TBE sites within the** ***ABCD2*** **promoter decreased** ***β*** **-catenin/TCF-4 mediated promoter activity in Huh7 cells.** Huh7 cells were cotransfected with the indicated expression plasmids along with either wild-type (WT) 800-Luc reporter plasmid containing wild-type (WT) TBE sequences or 800-Luc reporter plasmids containing the mutation of one or both TBE sites of the *ABCD2* promoter (mTBE1, mTBE2, and DmTBE, respectively). Mutated sequences are underlined (lower panel). At 36 h after transfection, cells were harvested, and luciferase activities were determined. The amount of DNA in each transfection was kept constant by adding an appropriate amount of pcDNA3 empty vector. Data represent the mean (± SEM) of triplicate experiments. *p<0.001.
(TIF)

Figure S2 **Contribution of the two TBE sites within the** ***ABCD2*** **promoter to** ***β*** **-catenin/TCF-4-mediated transcriptional activation in fibroblast cells from an X-ALD patient.** Primary fibroblasts isolated from an X-ALD patient were electroporated with the indicated expression plasmids along with either wild-type (WT) 800-Luc reporter plasmid containing the wild-type (WT) sequences or the double mutant (D-Mut) 800-Luc reporter plasmid containing mutations in the two TBE sites of the *ABCD2* promoter. The mutated sequences are underlined (lower panel). At 36 h after electroporation, cells were harvested, and luciferase activities were determined. The amount of DNA in each transfection was kept constant by adding an appropriate amount of pcDNA3 empty vector. Data represent the mean (± SEM) of triplicate experiments. *p<0.001.
(TIF)

Figure S3 ***β*** **-catenin and TCF-4 increase the transcriptional activity of** ***ABCD2*** **in Huh7 cells.** Huh7 cells were cotransfected with *ABCD2* promoter reporter (800-Luc) plasmids together with the indicated expression plasmids. At 36 h after transfection, cells were harvested, and luciferase activities were determined. The amount of DNA in each transfection was kept constant by adding an appropriate amount of pcDNA3 empty vector. Data represent the mean (± SEM) of triplicate experiments.
(TIF)
