# Supplementary figures and images for: ABCD2 Is a Direct Target of β-Catenin and TCF-4: Implications for X-Linked Adrenoleukodystrophy Therapy
Source: PLoS One. 2013 Feb 21;8(2):e56242. doi: 10.1371/journal.pone.0056242 (PMC3578850; doi:10.1371/journal.pone.0056242)

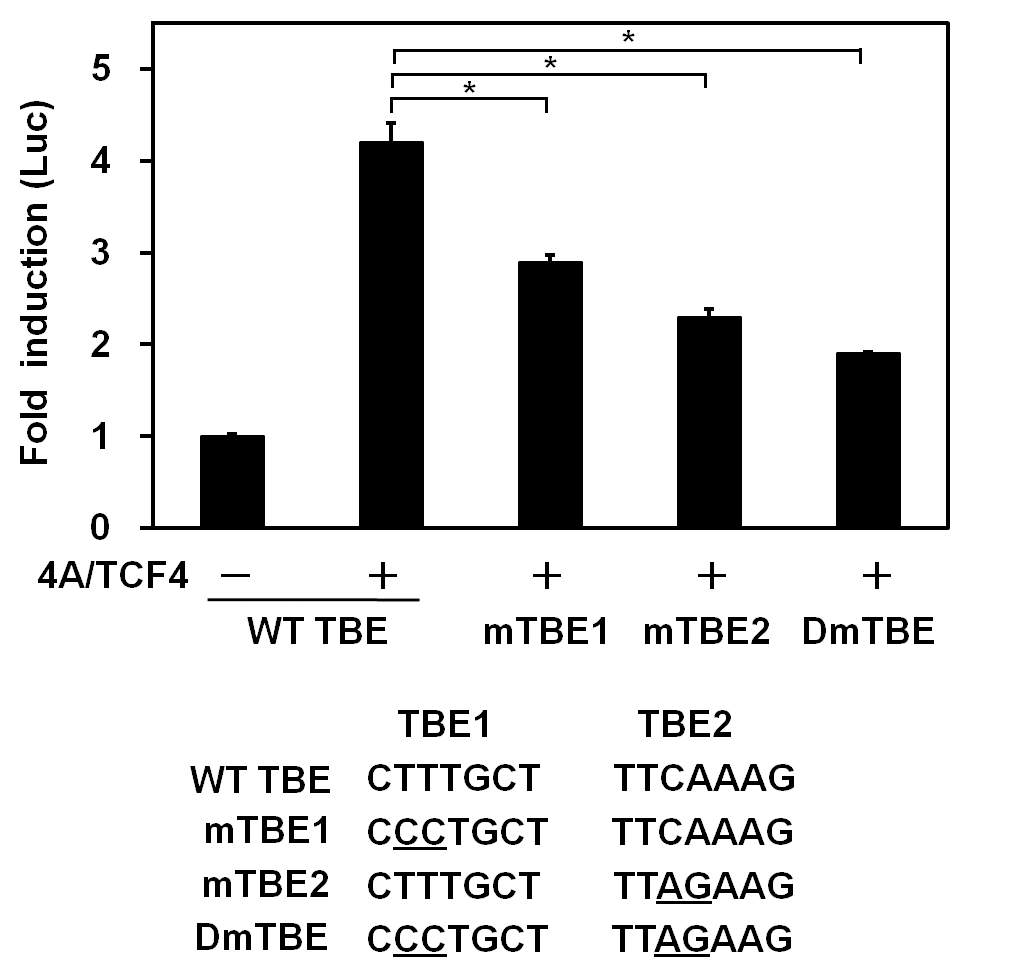

Supplement: Figure S1 — Mutation of TBE sites within the ABCD2 promoter decreased β -catenin/TCF-4 mediated promoter activity in Huh7 cells. Huh7 cells were cotransfected with the indicated expression plasmids along with either wild-type (WT) 800-Luc reporter plasmid containing wild-type (WT) TBE sequences or 800-Luc reporter plasmids containing the mutation of one or both TBE sites of the ABCD2 promoter (mTBE1, mTBE2, and DmTBE, respectively). Mutated sequences are underlined (lower panel). At 36 h after transfection, cells were harvested, and luciferase activities were determined. The amount of DNA in each transfection was kept constant by adding an appropriate amount of pcDNA3 empty vector. Data represent the mean (± SEM) of triplicate experiments. *p<0.001. (TIF) [file pone.0056242.s001.tif]

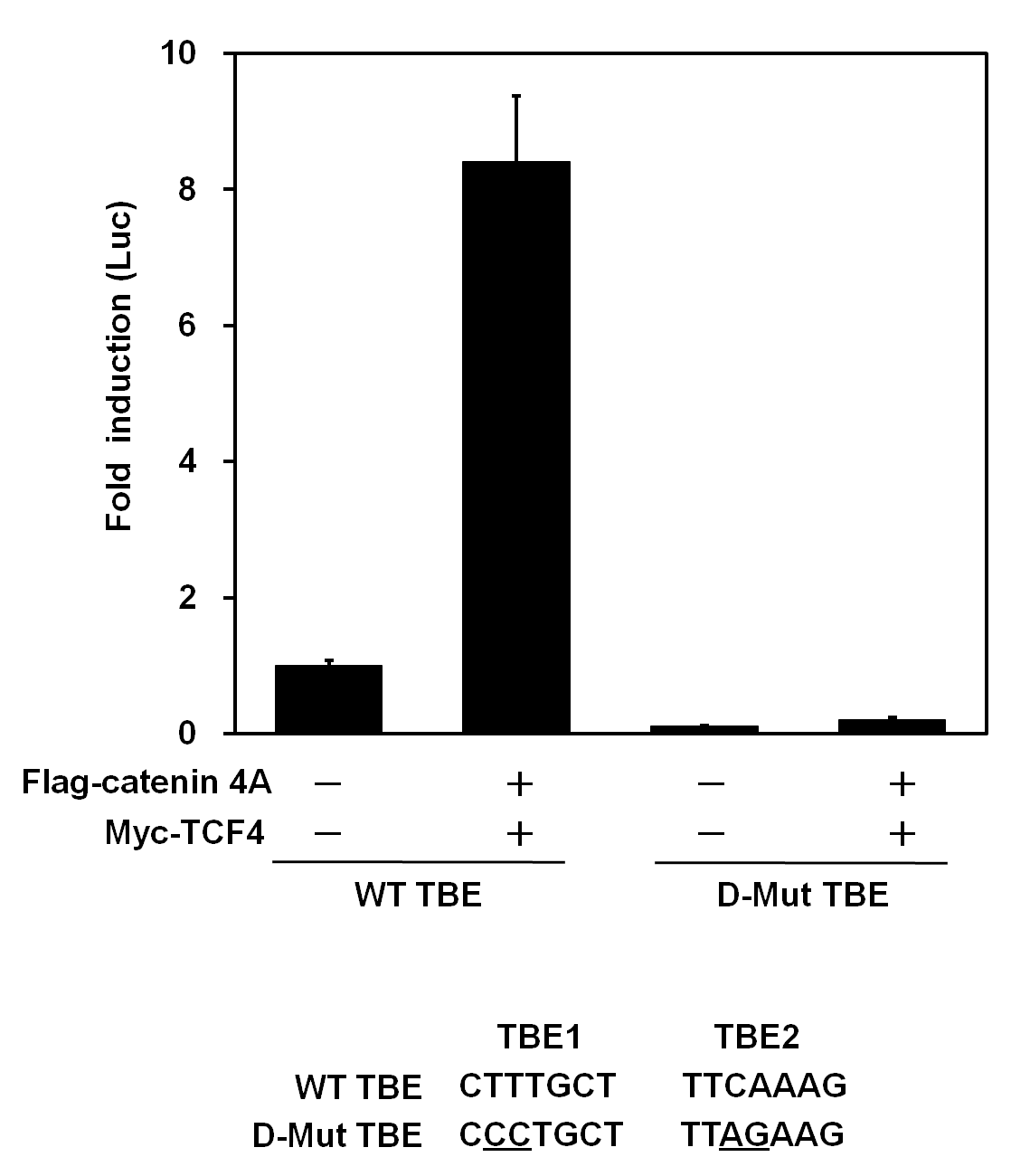

Supplement: Figure S2 — Contribution of the two TBE sites within the ABCD2 promoter to β -catenin/TCF-4-mediated transcriptional activation in fibroblast cells from an X-ALD patient. Primary fibroblasts isolated from an X-ALD patient were electroporated with the indicated expression plasmids along with either wild-type (WT) 800-Luc reporter plasmid containing the wild-type (WT) sequences or the double mutant (D-Mut) 800-Luc reporter plasmid containing mutations in the two TBE sites of the ABCD2 promoter. The mutated sequences are underlined (lower panel). At 36 h after electroporation, cells were harvested, and luciferase activities were determined. The amount of DNA in each transfection was kept constant by adding an appropriate amount of pcDNA3 empty vector. Data represent the mean (± SEM) of triplicate experiments. *p<0.001. (TIF) [file pone.0056242.s002.tif]

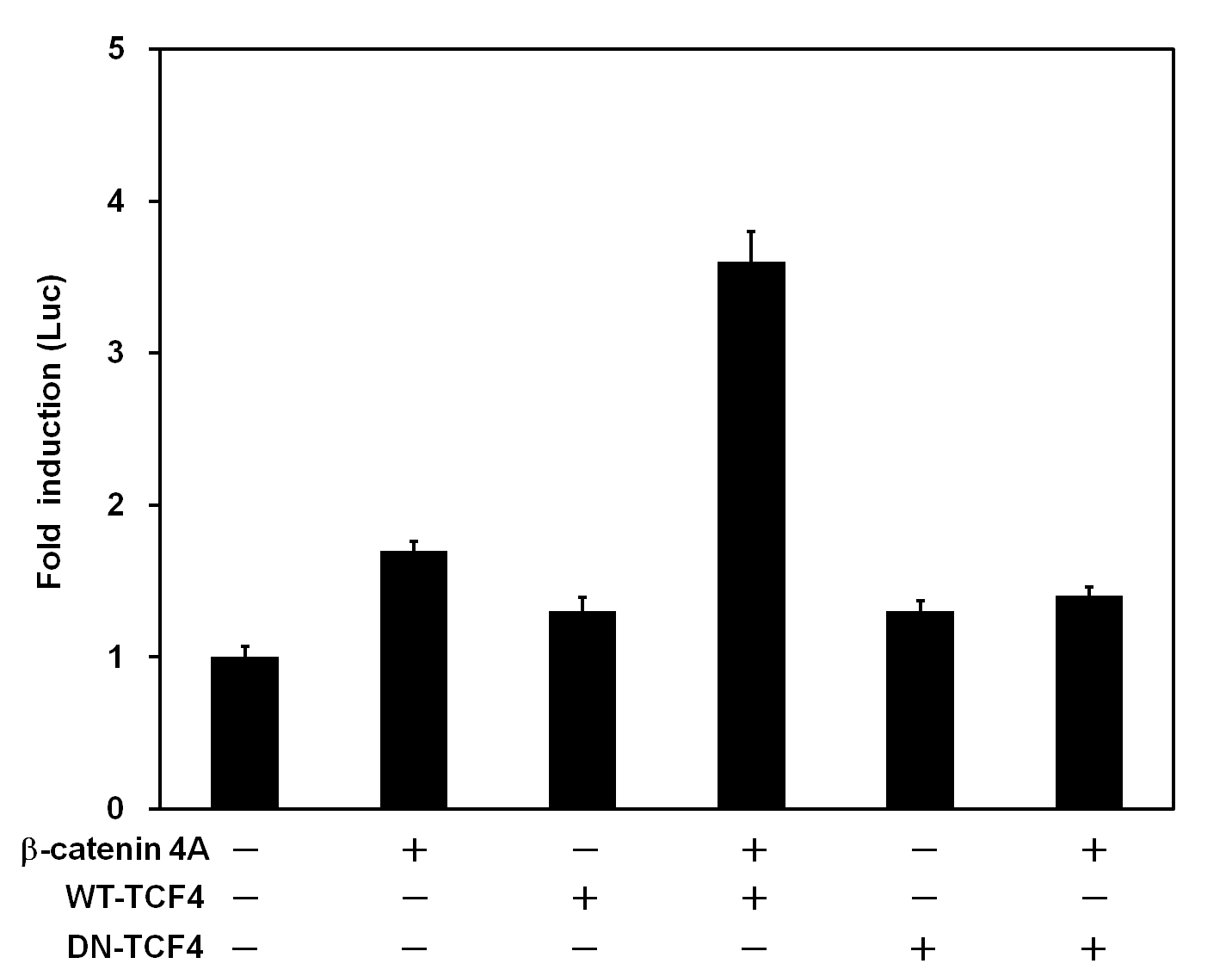

Supplement: Figure S3 — β -catenin and TCF-4 increase the transcriptional activity of ABCD2 in Huh7 cells. Huh7 cells were cotransfected with ABCD2 promoter reporter (800-Luc) plasmids together with the indicated expression plasmids. At 36 h after transfection, cells were harvested, and luciferase activities were determined. The amount of DNA in each transfection was kept constant by adding an appropriate amount of pcDNA3 empty vector. Data represent the mean (± SEM) of triplicate experiments. (TIF) [file pone.0056242.s003.tif]
